# The tale of histone modifications and its role in multiple sclerosis

**DOI:** 10.1186/s40246-018-0163-5

**Published:** 2018-06-22

**Authors:** Hui He, Zhiping Hu, Han Xiao, Fangfang Zhou, Binbin Yang

**Affiliations:** Department of Neurology, 2nd Xiangya Hospital, Central South University, No 139, Renmin Road, Changsha, Hunan Province China

**Keywords:** Histone modifications, Multiple sclerosis, Immune-mediated injury, Myelin destruction, Neurodegeneration

## Abstract

Epigenetics defines the persistent modifications of gene expression in a manner that does not involve the corresponding alterations in DNA sequences. It includes modifications of DNA nucleotides, nucleosomal remodeling, and post-translational modifications (PTMs). It is becoming evident that PTMs which act singly or in combination to form “histone codes” orchestrate the chromatin structure and dynamic functions. PTMs of histone tails have been demonstrated to influence numerous biological developments, as well as disease onset and progression. Multiple sclerosis (MS) is an autoimmune inflammatory demyelinating and neurodegenerative disease of the central nervous system, of which the precise pathophysiological mechanisms remain to be fully elucidated. There is a wealth of emerging evidence that epigenetic modifications may confer risk for MS, which provides new insights into MS. Histone PTMs, one of the key events that regulate gene activation, seem to play a prominent role in the epigenetic mechanism of MS. In this review, we summarize recent studies in our understanding of the epigenetic language encompassing histone, with special emphasis on histone acetylation and histone lysine methylation, two of the best characterized histone modifications. We also discuss how the current studies address histone acetylation and histone lysine methylation influencing pathophysiology of MS and how future studies could be designed to establish optimized therapeutic strategies for MS.

## Background

Epigenetic modifications is the ensemble of mechanisms of concurrent chromatin modification to modulate global patterns in gene expression and phenotype in a heritable manner, without affecting the DNA sequence itself, which can be classified into DNA modifications (methylation and hydroxymethylation) [1], (PTMs) [2], exchange of histone variants (e.g., H1, H3.3, H2A.Z, H2A.X) [3], and as non-coding RNA [4]. Unlike genes, which remain largely stable across a person’s lifetime, the epigenome is highly dynamic. To get a better understanding of how this works, in 2008, the NIH invested in an exploration of the epigenome, launching its Roadmap Epigenomics Mapping Consortium. The project set out to produce a public resource of human epigenomic data that would help fuel basic biology and disease research.

Up to now, the most intensely studied epigenetic modification is DNA methylation; however, the most diverse modifications are on histone proteins. There are at least eight distinct types of modifications found on histones, including acetylation, methylation, phosphorylation [5], ubiquitylation [6], sumoylation [7], ADP ribosylation [8], deamination [9], and prolineisomerization [10]. Histone acetylation and histone methylation are among the most prevalent histone modifications. Researches in the last decades has greatly advanced our knowledge of not only histone modification but also modification of non-histone proteins, providing functional diversity of protein-protein interactions, as well as protein stability, localization and enzymatic activities. Given the complexity of the topic, in the current review, we will concentrate specifically on histone acetylation and histone lysine methylation, of which we now have the most information.

MS is a chronic debilitating disease that affects the brain and spinal cord. Familial clustering is one of important characteristics of MS, suggesting that a hereditary factor involved in determining the risk of MS [11]. However, twin studies showed that monozygotic twins are genetically identical, but a monozygotic twin whose co-twin afflicted with MS has only 25% risk of developing the disease [12]. This suggests that the disease phenotype results from genetic code itself, as well as the regulation of this code by other factors. Increasing evidence suggests that epigenetic modifications may hold the keys to explain the partial heritability of MS risk [13]. In addition, it is believed that epigenetic mechanisms mediate the response to many environmental influences including geographic location, month of birth, Epstein-Barr virus (EBV) infection [14], smoking [15], and latitude/vitamin D [16], which ultimately affect disease development. In this review, we propose a view of MS pathogenesis that specifically involves histone modulations.

## Post-translational histone modifications

**Fig. 1:**
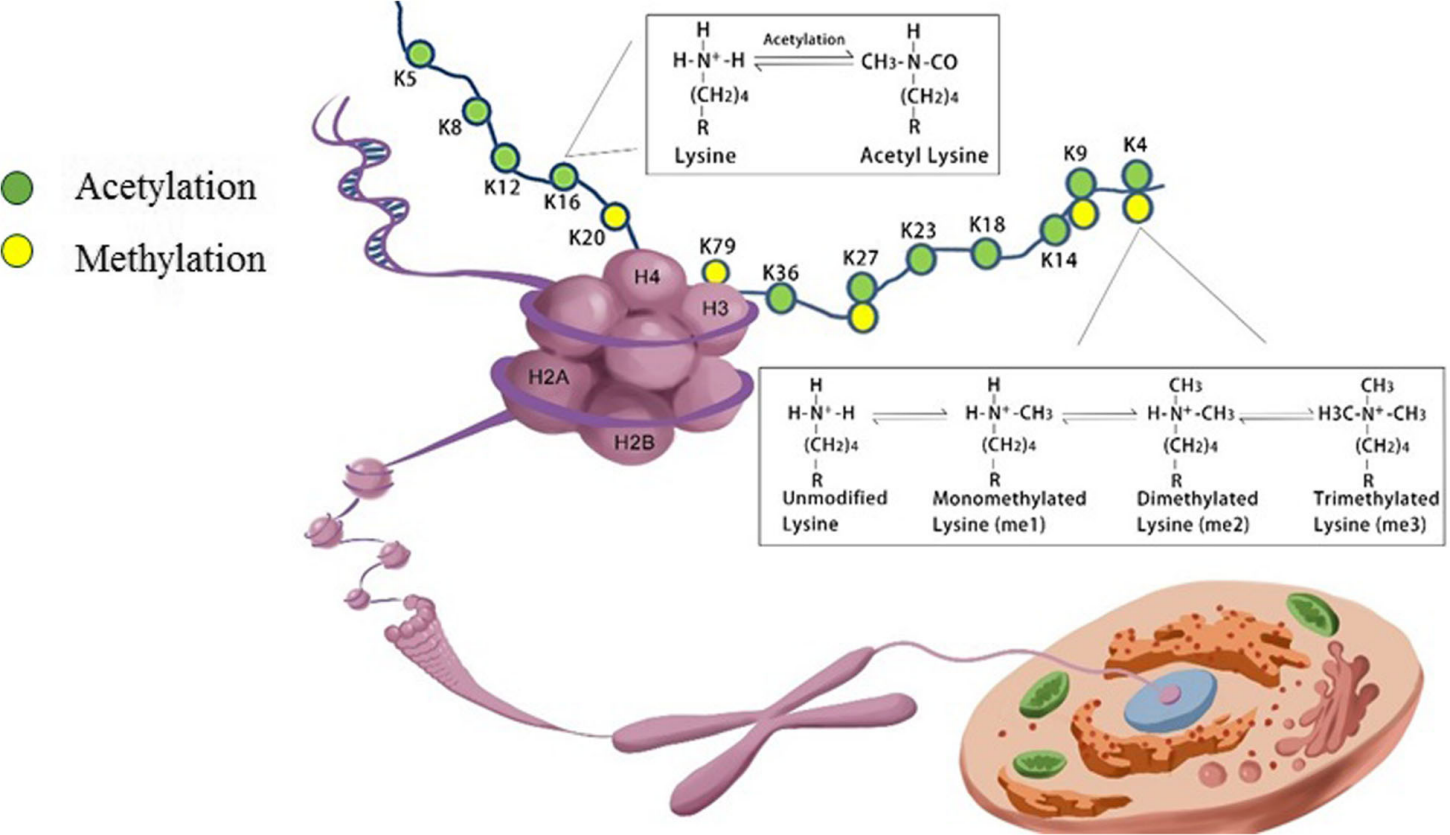
Schematic presentation of a nucleosome. A nucleosome functions as the fundamental packing unit of chromatin, with a stretch of double-stranded DNA wrapped around a histone octamer of two H2A–H2B dimers and a (H3–H4) 2 tetramer. Different possible histone modifications (mainly acetylation and methylation) at core histones and the processes of the modifications are shown

Histones are among the most highly conserved proteins that act as building blocks of the nucleosome, the fundamental structural and functional unit of chromatin. The nucleosome is an octamer, which is wrapped by147 bp of DNA, consisting of two copies of four core histone (H) H2A, H2B, H3, and H4 around, tied together by linker histone H1 [17]. These five classes of histone proteins, bearing over 60 different residues, constitute the major protein components of the chromatin and provide a tight packing of the DNA. Meanwhile, the histones contain a flexible N-terminus, often named the “histone tail” [17], which can undergo various combinations of PTMs, dynamically allowing regulatory proteins access to the DNA to fine tune almost all chromatin-mediated processes including chromatin condensation, gene transcription, DNA damage repair, and DNA replication [18] (Fig. 1). Transcriptionally active and silent chromatin is characterized by distinct post-translational modifications on the histones or their combinations. H3K27ac and H3K4me1 are associated with active enhancers [19], and high levels of H3K4me3 and H3 and H4 acetylation are found at the promoters of active genes [20, 21]. The bodies of active genes are enriched in H3 and H4 acetylation [22], H3K79me3 [23], H2BK120u1, and a progressive shift from H3K36me1 to H3K36me3 between the promoters and the 3′ ends [24]. The methylation of H3K27 and H3K9 have emerged as hallmarks of repressive chromatin and are often found at silent gene loci. H3K27me3 are associated with the formation of facultative heterochromatin, whereas H3K9me2/3 has important roles in the formation of constitutive heterochromatin [25]. H4K20m3 is a novel hallmark of pericentric heterochromatin, whereas H4K20m1 regulates transcription both positively as well as negatively [26], suggesting that specific histone modifications can have dual functions. There are many combinations of modifications that are either occurring together or mutually exclusive, suggesting crosstalk between these marks. Combinations of PTMs, thus, may be associated with transcription in a manner that was not simply related to their individual effects. For example, Fischer et al. indicated that single-code histone acetylation, in particular H3 acetylation (H3ac), are better predictors of increased transcript levels than domains containing further modifications [27]. Single-code H3K4dimethylation (H3K4m2) or its combination with H3K4 tri-methylation (H3K4m3) showed no positive correlation with transcript levels [27]. It is interesting given that H3K4m3 is known to be associated with transcription-start sites of actively transcribed genes. The results from Fischer and his colleague suggested that H3K4me3 is actually not an optimal marker of active promoters and that the activating effect mainly results from its frequent colocalization with acetylations [27].

Histone proteins can undergo post-translational modifications by “writers” and “erasers,” a set of enzymes responsible for the deposition and removal of the chemical modifications. Through different combinations and patterns of histone PTMs, they can form the “histone code” [28]. Then, how are these codes interpreted? There are several mechanisms that are not mutually exclusive. First, direct nucleosome-intrinsic effects, particularly by neutralization or addition of charge, PTMs weaken histone-DNA interaction and enable generation of a stably remodeled nucleosome with increased mobility [29]. Such conventional allosteric regulation usually relies on a highly specialized population of molecular interactions [30]. Second, in direct nucleosome-extrinsic effects, H4K16 has been demonstrated to be such a unique histone tail, the acetylation of which impedes the higher-order chromatin formation as a result of its modulation of internucleosomal contacts [31]. Third, the emerging effector-mediated paradigm posits that histone PTMs are “read” by protein modules termed as effectors, which translate them into patterns of gene activation or repression recruiting transcriptional or chromatin-remodeling machinery [30]. In the past decade, a wealth of conserved protein-interaction domains has been characterized as histone effectors, which recognize and bind histone PTMs specifically in a modification- and site-specific way. By covalent combinations of PTMs for binding, modified histone tails may function as integrating platforms for different chromatin-associated complexes, permitting them to receive inputs from upstream signaling cascades and transmit them to the downstream effectors [32].

## Histone acetylation

Histone acetylation has been shown to be reversible. The N-terminal domains of histones bear a dozen of lysine residues subject to acetylation, with the exception of a lysine within the globular domain of H3K56, which was found to be acetylated in human by GCN5 [33]. This K residue is facing towards the major groove of the DNA within the nucleosome, so it is in good position to modulate nucleosome assembly by altering histone-DNA interactions when acetylated [34].

### Readers of acetyl-lysines

The combinatorial effect of histone acetylation can be deciphered by two distinct, yet overlapping mechanisms—direct and effector-mediated readout mechanisms. In the direct mechanism, histone acetylation neutralizes the positive charge on lysine residues, thus destabilizing the DNA-histone interaction [35]. This results in an open, loosely packed chromatin structure (euchromatin) and consequently allows access for specific transcription factors and the general transcription machinery [31].

Alternatively, histone lysine acetylation marks may be interpreted indirectly via the intermediacy of effectors, which also generally serve to enhance transcriptional activation. The recognition of lysine residues is primarily initiated by bromodomains (BRD) [36]. In general, isolated BRD has been shown to bind to acetylated histones with relatively low affinity and relatively poor selectivity [37], yet, in the presence of multivalent binding, the specificity and affinity are frequently increased. For example, the tandem BRDs of human TATA-binding protein-associated factor-1 (TAF1) binds to multiple acetylated histone H4 peptides with increased affinity, each BRD engaging one acetyl-lysine mark in the same peptide [38]. In principle, the apposition of two BD modules rigidly confined in a relative orientation creates surfaces that are complementary to the spatial distributions of their substrates in chromatin. Therefore, the distances between discrete interactions become additional determinants in dictating specificity [38]. More recently, it has been demonstrated that two acetylated lysine residues might be simultaneously recognized by the same BRD module with significantly increased affinity. For example, a single binding pocket of BD1 of BRDT accommodates both acetyl-lysines of H4K5acK12ac and H4K8acK16ac peptides in a wider hydrophobic pocket, showing much stronger affinity than binding to either mark individually [39]. Moreover, the acetylated histone recognition by BD1 is complemented by a novel BRD-DNA interaction [40]. Simultaneous DNA and histone recognition enhances BRD’s nucleosome binding affinity, specificity, and ability to localize to and compact acetylated chromatin [40].

### Writers and erasers of acetylation

KATs, formally named as histone acetyltransferaces (HATs), can be generally classified into two categories based on subcellular localization. Type A KATs are located in the nucleus, involved in the acetylation of histones in chromatin, whereas type B KATs, predominantly cytoplasmic, acetylate newly translated histones to facilitate their transfer to the chromatin assembly factors [41]. In eukaryotes, the majority of canonical type A KATs has been grouped into three major families including p300/CBP, GCN5/PCAF, and MYST proteins [42] (Table 1). Two subfamilies of histone deacetylases (HDACs) have been identified in humans so far—Zn2+-dependent (classes I, II, and IV) and Zn2+-independent and NAD-dependent (class III). Generally speaking, class I HDACs are ubiquitously expressed and exhibit strongest enzymatic activity. Class II HDACs have sequence similarity to the yeast Hda1 protein which seems to be expressed in a more cell-specific manner [43]. They possess unique 14-3-3 binding sites at their N-termini. Following phosphorylation, the N-terminal regions recruit 14-3-3, with consequent export of the HDAC/14-3-3 complex from the nucleus to the cytoplasm [44, 45]. Thus, phosphorylation of class II HDACs provides a mechanism for coupling external signals to the genome. The class III HDACs, or sirtuins, display NAD+-dependent deacetylase activity and may specifically interact with and modify dozens of distinct substrates in various the biological processes.

**Table 1:** Enzymatic mechanisms used for histone acetylation

Canonical members of KAT	Former name in human	Histone protein acetylated	Mechanism of catalysis
P300/CBP family			
*KAT3*			Hit-and-run
KAT3A	CBP	H2A, H2B	
KAT3B	P300	H2A, H2B	
GCN5 family			
*KAT2*			KAT/Ac-CoA/substrate ternary complex
KAT2A	GCN5	H3, H4,H2B	
KAT2B	PCAF	H3	
MYST family			
*KAT5*	Tip60	H4, H2AZ, H2AX	Ping-pong mechanism or ternary mechanism
*KAT6*			
KAT6A	MOZ/MYST3	H3	
KAT6B	MORF/MYST4		
KAT7	HBO1/MYST2	H4	
KAT8	MOF/MYST1	H4	

## Histone lysine methylation

Histone methylation occurs at lysine and arginine residues. In this review, we only focus on histone lysine methylation due to its prominence and its array of well-established roles in epigenetic gene control and chromatin domains organization. Histone lysine methylations have been found on a range of lysine residues in various histones, including K4, K9, K27, K36, and K79 residues in histone H3, K20 in histone H4, K59 in the globular domain of histone H4 [46], and K26 in histone H1B [47]. Instead of influencing the net charge of the histone tails, methylation of histone tails contributes to regulation of the transcriptional activity by functioning as a recognition template to recruit effector proteins to local chromatin [48]. Thus, histone lysine methylation can be associated with either activation or repression of transcription ultimately determined by the effectors. When compared with acetylation, histone lysine methylation is a relatively stable modification with a generally low turnover [49]. Moreover, methylation is controlled by histone methyltransferases (KMTs) and demethylases (KDMs) that possess strong substrate specificity (Table 2) (Table 3).

**Table 2:** Substrate specificity of KMTs and KDMs

		H3K4			H3K9			H3K27			H3k36			H3K79			H4k20	
	Me1	Me2	Me3	Me1	Me2	Me3	Me1	Me2	Me3	Me1	Me2	Me3	Me1	Me2	Me3	Me1	Me2	Me3
WRITERS	KMT2A	KMT2A	KMT2A	KMT1C	KMT1C	KMT1A		KMT1C		KMT3B	KMT3B	KMT3A	KMT4	KMT4	KMT4		KMT3B	
	KMT2B	KMT2B	KMT2B	KMT1D	KMT1D	KMT1B					KMT3C					KMT5A	KMT5B	KMT5B
	KMT2C	KMT2C	KMT2C			KMT1E											KMT5C	KMT5C
	KMT2D	KMT2D	KMT2D			KMT1F			SMYD3									
	KMT2E	KMT2E	KMT2E				KMT6A (?)	KMT6A	KMT6A									
	KMT2F	KMT2F	KMT2F				KMT6B (?)	KMT6B	KMT6B									
	KMT2G	KMT2G	KMT2G															
	KMT3C																	
	KMT3D (?)	KMT3D (?)	KMT3D (?)															
		KMT3E	KMT3E															
	KMT7																	
				KMT8														
ERASERS	KDM1A	KDM1A		KDM1A	KDM1A	KDM2B		KDM6	KDM6	KDM2	KDM2							
					KDM3						KDM4	KDM4						
					KDM4	KDM4					KDM8							
		KDM5	KDM5	KDM7B	KDM7B		KDM7B	KDM7B								KDM7A		KDM7C
																KDM7B

**Table 3:** Histone methyltransferases and demethyltransferases

Writers		
	KMT1	
SUV family	KMT1A	SUV39H1
	KMT1B	SUV39H2
	KMT1C	G9a
	KMT1D	GLP
	KMT1E	SETDB1
	KMT1F	SETDB2
	KMT2	
MLL family	KMT2A	MLL1
	KMT2B	MLL2
	KMT2C	MLL3
	KMT2D	MLL4
	KMT2E	MLL5
	KMT2F	SET1A
	KMT2G	SET1B
	KMT2H	ASH1
	KMT3	
NSD family	KMT3A	SETD2
	KMT3B	NSD1
	KMT3F	NSD3
	KMT3G	NSD2
SMYD family	KMT3C	SMYD2
	KMT3D	SMYD1
	KMT3E	SMYD3
	KMT4	DOT1L
	KMT5	
	KMT5A	SET8
	KMT5B	SUV420H1
	KMT5C	SUV420H2
	KMT6	
	KMT6A	EZH2
	KMT6B	EZH1
	KMT7	SET7/9
	KMT8	PRDM2/RIZ1
Erasers		
	KDM1	
	KDM1A	LSD1
	KDM1B	LSD2
	KDM2	
FBXL cluster	KDM2A	JHDM1A
	KDM2B	JHDM1B
	KDM3	
JMJD1 cluster	KDM3A	JMJD1A
	KDM3B	JMJD1B
	KDM3C	JMJD1C
	KDM4	
JMJD2 cluster	KDM4A	JMJD2A
	KDM4B	JMJD2B
	KDM4C	JMJD2C
	KDM4D	JMJD2D
	KDM5	
JARID1 Cluster	KDM5A	JARID1A
	KDM5B	JARID1B
	KDM5C	JARID1C
	KDM5D	JARID1D
	KDM6	
UTX/JMJD3 cluster	KDM6A	UTX/UTY
	KDM6B	JMJD3
	KDM7	
	KDM7A	JHDM1D
	KDM7B	JHDM1E
	KDM7C	JHDM1F
	KDM8	JMJD5

### Readers of methylysines

Chromodomain is the founding member of “readers” of histone methyllysine [50], Besides the well-known methy-lysine-binding family of chromodomain, a large family of reader proteins including Tudor, MBT, PWWP, plant homeodomain (PHD) finger, Ankyrin repeats, and WD repeats make up the so-called Royal family [51, 52]. Three elements determine the strength and specificity of a particular methylated lysine reader. The foremost trait of the methyllysine readers is the presence of an aromatic cage structure in their binding to methyllysines, consisting two to four aromatic residues. The exact composition and size of the pocket make the readers selective in recognizing mono-, di-, or trimethylated state of lysine. Effectors for mono- and dimethylation tend to have a small keyhole-like cavity, which leads to steric hindrance to limit accessibility of a higher methylation state [53]. In contrast, the binding pockets of effectors for di- and trimethylation are wider and more accessible, which may also lower the stringency in the discrimination preferences [53]. Typically two ways are involved in the recognition of methyl states. At some lysines, selective effector is recruited to a specific methylation state. For instance, Pdp1 binds to H4K20me1 to facilitate chromatin maturation, whereas 53BP1 in mammals and Crb2 in fission yeast selectively bind the H4K20me2, required for DNA damage checkpoint activation [54]. At other sites, methyl states only influence the binding affinity of the same histone-methyl-lysine-binding proteins. For example, Rpd3S preferentially binds K36me2 and K36me3, with K36me3 displaying the highest affinity. By contrast, the affinity of K36me1 to Rpd3S is much lower, similar to that of the unmodified ones [55]. Secondly, interaction with flanking sequence may impart an additional layer of specificity for a particular methylated lysine. Free histone peptides are usually unstructured in aqueous solution. On binding, they adopt a β-sheet conformation, with extensive contacts with the flanking sequence of the readers [56]. This pairing interaction not only contributes to the overall robustness but also provides structural basis for functional specificity [53]. At last, methyllysines are located close to the end of a histone peptide; upon binding, the histone termini can be buried snugly into a shallow pocket, which greatly facilitates the overall affinity [53].

### Writers and erasers of histone lysine methylation

KMTs catalyze methylation of lysine residues with high site- and methyl-level specificity (Table 2). In the last decades, numerous KMTs have been identified and crystallized, which use *S*-adenosylmethionine (SAM) as a methyl group donor [57]. Except for KMT4/DOT1L, all known KMTs contain a conserved SET domain harboring the enzymatic activity [58]. Based on the similarities in the sequence within and around the catalytic SET domain, as well as homology to other protein modules and their domain architectures, SET-containing KMTs have traditionally been categorized into distinct subfamilies [59].

Histone lysine methylation was previously considered static and enzymatically irreversible until the first histone KDM—LSD1/KDM1A identified by Shi et al. [60], which changed our view of histone methylation regulation and ushered in the identification of numerous histone demethylases. Subsequent to the discovery of KDM1A, a new class of KDM enzymes which comprises the JmjC domain-containing protein was discovered. While KDM1A is unable to catalyze the dimethylation of trimethylated lysine residues owing to its requirement for imine formation for catalytic activity, the JmjC-driven demethylase have demethylation activity for mono-, di-, and trimethylated histone lysine residues. Indeed, most of the JmjC histone demethylases identified so far are capable of efficiently catalyzing demethylation of trimethylated lysines, and in most cases, they preferentially bind the trimethylated substrates [61, 62].

## Histone modifications in MS

A core of pathogenetic functions common to both the immune and neurodegenerative processes of MS has been characterized by deregulation of MS-risk genes and resulting dysfunction of their encoding proteins [63]. Epigenetic transcription-regulating mechanisms in nucleated cells including cells of the CNS have been widely accepted. Therefore, MS-specific alterations in epigenetic regulation of chromatin may play a central role in gene expression and be essential for the initiation and development of MS. Among which, histone modification is an important epigenetic mechanism.

### Histone modifications in MS susceptibility

Twin studies have established that susceptibility to MS is partly genetic. One family of major histocompatibility complex (MHC) genes, the human leukocyte antigen (HLA) alleles, has identified as a genetic determinant for MS [64]. In particular, carriage of HLA-DR/DQ serotype has been identified as a major MS risk allele. Notably, expression of HLA-DR has been shown to be suppressed by HDAC1 [65], which suggests that MS susceptibility loci have histone regulation links.

### Histone modifications in autoimmunity-related mechanisms

The hallmark of MS and experimental autoimmune encephalomyelitis (EAE) is that myelin injury and axonal damage driven by an immune-mediated inflammatory response begins at disease onset. Autoreactive myelin-specific CD4+ T cells are believed to play a crucial pathogenic role [66]. Upon encountering myelin antigen, antigen-presenting cells (APCs) acquire a mature phenotype and migrate to lymph nodes where they present exogenous antigens to naïve CD4+ T cells. Naive CD4+ T cells may then differentiate into diverse functional subsets, including the T helper (Th) 1, Th2, Th17 cells, and Treg cells [67]. Once activated, CD4+ T cells are translocated into the CNS by crossing the brain-blood barrier (BBB) and then are reactivated by resident APCs (such as microglia) [68], which in turn initiate the recruitment of other inflammatory cells, resulting in demyelination and axon injury. While interferon-γ (IFN-γ)-associated Th1 and interleukin-17 (IL-17)-associated Th17 cells are considered to lead to disease progression and worsening of symptoms, IL-4-associated Th2 and transforming growth factor-β (TGF-β)-associated Treg have been indicated to associate with inflammation reduction and improvement of symptoms in MS patients [69].

It is widely accepted that the activation of CD4+ autoreactive T cells and their differentiation into a Th1 or Th17 phenotype are crucial events in the initial steps of MS, though many studies have shown that monocytes and monocyte-derived macrophages are also the primary cell types responsible for cellular pathology and tissue damage. In MS pathology, activated monocytes, which facilitate the migration of T cells across the blood-brain barrier (BBB), largely represent the inflammatory infiltrate [70]. Knowledge on the features of blood monocytes in MS, however, are little understood. Circulating monocytes, as an important source of cytokines, have been hypothesized to play a key role in regulating crucial immune functions. The M1/M2 paradigm is currently used to categorize the monocyte/macrophage functions [71], and M1/M2 macrophage balance polarization governs the fate of an organ in inflammation. Generally, M1 monocytes/macrophages are generally characterized by an IL-12hi, IL-23hi, tumor necrosis factor (TNF)-αhi, and IL-10lo phenotype, which produce abundant reactive oxygen species and shift the immune response towards a Th1 profile [72]. M2 monocytes/macrophages typically have IL-12lo, IL-23lo, TNF-αlo, and IL-10hi responses to stimulation, which are thought to drive Th2 responses [73].

HDACs have been shown to be closely tied to regulation of CD4+ T cells differentiation and various cytokines production through regulating the changes in chromatin structure which then influence gene expression. Correspondingly, HDAC inhibitors have also been demonstrated to elicit control over the immune response, which in turn suppress systemic and local inflammation [74]. Several recent studies have shown the potential for the use of HDAC inhibitor therapy to inhibit the proliferative response of CD4+ T cells and abrogated IFN-γ production [75]. A growing literature indicated that HDAC inhibitors inhibit the proinflammatory cytokine IL-2 expression, which is secreted by Th1 cells, and IL-2 mediated gene expression as well. Moreover, HDAC inhibitors reduce macrophage production of pro-demyelinating cytokines involved in T helper (Th) cell differentiation, including IL-12, IL-6, and TNF-α. Consequently, HDAC inhibitors cause a Th1 to Th2 dominance shift [76], and expanding Tregs, which by virtue of its immunosuppressive role, may help ameliorate MS.

Actually, dysregulated Th cell responses are not unique for MS pathology, but also a characteristic of a wide variety of several other inflammatory diseases, including inflammatory bowel disease, arthritis, diabetes, asthma, and allergies [77]. Therefore, compounds that inhibit HDACs, especially, class I, II, and IV enzymes, have been pursued as therapeutic agents for a wide range of inflammatory diseases. However, treating cells with HDAC inhibitors has also been shown to increase the expression of cytokines IL-10 [76], contributing to pro-humoral and protective role in EAE, which, in systemic lupus erythematosus (SLE) cells, actually downregulated expression of IL-10 and other anti-inflammatory cytokines [78]. The contrasting effects might reflect disease-specific effects of these compounds and further studies are needed.

It is suggested that chromatin remodeling, via histone lysine methylation, is mechanistically important in the acquisition of the M2-macrophage phenotype. Ishii et al. demonstrated that at the promoters of the M2 marker genes, H3K4me3 was significantly upregulated, whereas H3K27me2/3 was significantly decreased. Increased Jmjd3 contributes to the decrease of H3K27me2/3 marks and skews macrophages to an M2 phenotype [79]. Therefore, target gene regulation by histone Lysine methylation is a dynamic process that modulates inflammatory responses in the development of a variety of autoimmune diseases, including MS.

Recent studies demonstrated that KDM6 modulate immune functions by determining Th cell maturation and egress from the thymus [80], as well as CD4+ Th cell lineage differentiation [66], thereby significantly affecting immune responses in multiple biological systems. It is reported that Jmjd3 positively regulate the differentiation of Th17 cells, which play critical roles in proinflammatory reactions in autoimmue disorders, such as rheumatoid arthritis and systemic lupus erythematosis [81]. Jmjd3-deficient mice were demonstrated to be resistant to the induction of EAE [66]. Correspondingly, H3K27 demethylase-specific inhibitor GSK-J4 markedly inhibited Th17 cell differentiation in vitro [66]. However, another independent research demonstrated that while Th1 and Th17 differentiation were not affected, 10 or 25 nM GSK-J4 significantly increased differentiation of anti-inflammatory Treg cells in vivo, which could partly explain the beneficial effects of GSK-J4 on EAE. GSK-J4 promoted Treg differentiation was proposed to be dependent on its direct effect on the maturation status of dendrite cells (DCs). DCs, the professional APC, being the key players in maintaining immune tolerance, now have gained increasing attention [82]. Specifically, H3K27me3 demethylase activity would skew DC differentiation towards a tolerogenic phenotype [83]. Accordingly, through altering the permissive H3K4me3/repressive H3K27me3 ratio at specific gene promoters, GSK-J4 induced a tolerogenic phenotype on DCs and subsequently inhibited the development of EAE [83].

**Fig. 2:**
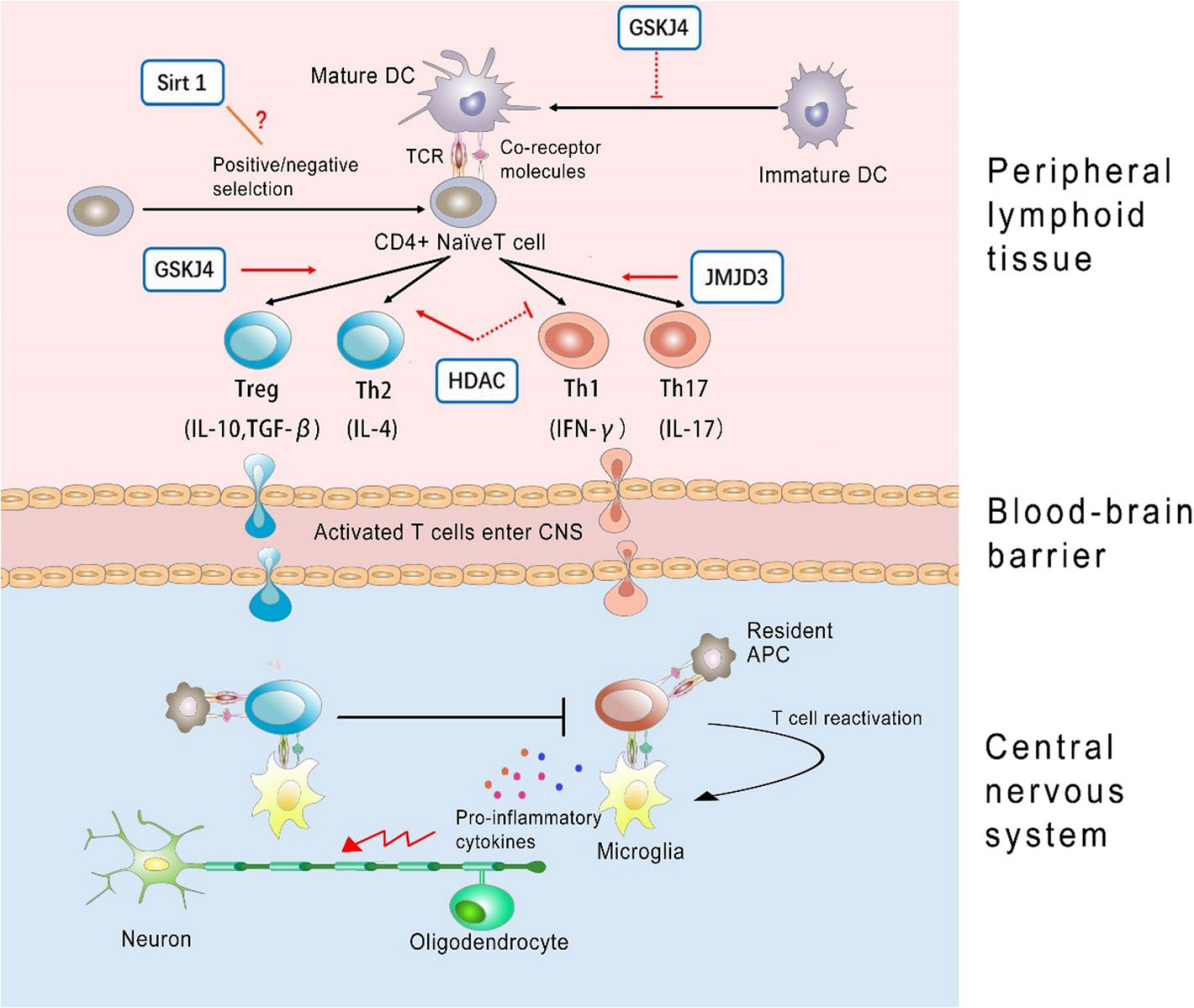
A model of immune mechanism in MS. Cascade of events possibly underlying autoimmunity-related demyelination in MS and putative mechanisms of action of histone-modifying enzyme inhibitors are demonstrated

Moreover, T cell anergy is thought to be a critical mechanism for preventing autoimmunity and failure of this tolerance mechanism causes MS [84]. The upregulated Sirt1 protein has been demonstrated to suppress T cell activation and lead to anergy induction in mice. Conversely, Sirt1 deficiency was reported to result in increased T cell activation and failed to maintain CD4+ T cell tolerance and increased susceptibility to EAE [85]. Mice with DC-specific deletion of SIRT1 showed remarkable resistance to EAE through enhanced IL-27 and IFN-β activation, two anti-inflammatory cytokines that negatively regulate Th17 cell differentiation [86]. These findings make the role of HDAC in MS quite controversial (Fig. 2).

### Histone modifications in myelin destruction

Another cardinal feature of multiple sclerosis is the failure of remyelination caused by impaired differentiation of endogenous oligodendrocyte progenitor cells (OPCs). Unlike other neuronal lineages, in the oligodendrocyte lineage, high levels of histone acetylation are important in undifferentiated progenitor cells [87], which favor the expression of transcriptional repressors of myelin gene expression. Increased histone H3 acetylation in oligodendrocytes is associated with high levels of transcriptional inhibitors of oligodendrocyte differentiation which subsequently might lead to impaired remyelination in patients with MS [88]. Conversely, histone deacetylation enables expression of an oligodendrocyte transcriptional profile during developmental myelination, as well as remyelination [87]. While a large number of oligodendrocytes with deacetylated histone was observed in early MS lesions, a shift towards high levels of histone acetylation has been detected in oligodendrocyte lineage cells within normal-appearing white matter (NAWM) in the brain of patients with chronic MS [89]. The data suggested negative correlations between histone deacetylation efficiency and duration of disease.

### Histone modifications in neurodegeneration

For decades, MS research has heavily focused on inflammatory white matter pathology. However, recent studies have discovered neurodegenerative components of the disease such as insidious axonal degeneration and neuronal atrophy, which seem to be the histopathological correlates of progressive clinical disability in MS patients [90]. Mitochondrial injury and subsequent energy failure are indicated as key factors in the induction of neurodegeneration. Betaine, a methyl donor, was found to be decreased in MS cortex, which was correlated with decreased H3K4me3 in neuronal (NeuN+) nuclei in MS cortex, in comparison to controls [91]. Mechanistic studies demonstrated that reduced methylation of H3K4me3 may result in the downregulation of oxidative phosphorylation genes and defects of respiratory chain enzymes in MS cortex [91]. A recent study showed that variant carriers of certain HDAC genes, including mitochondrial-related gene variants in SIRT4 and SIRT5, have been linked to more pronounced brain volume loss (atrophy) during the clinical course of MS [92]. These results indicate that the histone modifications might be centrally linked with neurodegenerative processes in MS.

## Potential treatment methods based on epigenetic mechanisms

Disturbance of transcriptional balance may promote dysregulation of immune system and neurodegeneration, both of which contribute to the clinical profile of MS. Animal model experiments support that deliberate epigenetic reprogramming for oligodendrocyte, immune cells, and neurons to perform properly may be a potential therapeutic strategy for MS.

There is a growing list of pharmacological agents that affect histone PTMs, among, which the most studied and used are histone deacetylase inhibitors (HDACi). For example, Camelo et al. showed that intraperitoneal administration of the HDACi, Trichostatin A (TSA) attenuated inflammation, reduced demyelination and axonal loss, and thus decreased disease severity in mice with spinal cord homogenate induced EAE [74]. The HDACi, vorinostat (SAHA), was shown to suppress DCs function and ameliorate EAE in C57BL/6 female mice [93]. VPA administration suppresses systemic and local inflammation to improve outcome of EAE in Lewis rats [94]. Likewise, curcumin, which inhibits the activity of KATs, has been shown to ameliorate EAE through suppression of inflammatory cells infiltration in the spinal cord [95]. As previously mentioned, systemic administration with the epigenetic drug GSK-J4 prevented the development of EAE in mice [83]. Thus, the inhibitors of histone deacetylation or demethylation may be promising agents for MS treatment. However, systemic use of HDACis negatively affects the generation of new myelin since histone deacetylation is important for progenitor cell differentiation into myelin-forming oligodendrocytes [96] and is critical for remyelination efficiency in adults [88], as we reviewed previously. The potential detrimental consequence on myelin might counteract the beneficial effects, thus cautioning against the use of broad inhibitors of histone deacetylases in MS. Therefore, more targeted therapy that specifically epigenetically modifies certain pathogenic loci need to be developed. In the recent years, the CRISPR-dCas9 system is poised to become the most promising targetable epigenome-editing tools. The results of two recent seminal studies have strongly supported the capability of epigenome editing by a CRISPR-Cas9 to activate or silence transcription by targeting histone PTMs [97, 98]. Moreover, CRISPR-dCas9 epigenome-editing approach has been demonstrated to produce long-lasting changes in expression of targeted genes both in vitro and in vivo. Its simplicity and efficiency may facilitate the clinical application of this technology by avoiding repetitive or chronic administration. However, the research on CRISPR-mediated technology is still in its early stage, and it is important to continue to probe for its feasibility and safety for clinical purposes. An additional challenge for treating MS with these inhibitors is the lack of specificity, which would cause a relatively high risk of adverse effects. Correspondingly, successful epigenetic therapy would be the tissue specificity of the therapeutic effect. Receptor-coated nanoparticles or microvesicles as highly effective drug carriers pertaining to BBB may hold great promise in MS therapy. Several studies have recently demonstrated that treatment of mice with nanoparticles effectively decreased EAE progression [99]. Collectively, translational use of epigenetics might offer hope for a new class of therapeutics to treat MS and the development of targeted epigenetic therapies open new avenues for effective personalized treatment of patients with MS.

## Conclusion

MS is the most prevalent autoimmune disease with highly variable clinical course and disease progression, in which the main common pathogenetic pathway involves an immune-mediated cascade [100]. Recently, huge steps have been made in the field of MS immunotherapy. Moreover, emerging evidence has shed light on the epigenetic mechanisms contributing MS. Several epigenetic drugs which are in clinical trials or under investigation in human diseases have been proven to have immunomodulatory effects [101]. In addition, other expected changes also may occur in response to epigenetic treatment. In particular, histone PTMs in regulation of myelination and degeneration gene associated with MS and amelioration of EAE symptoms by drugs with PTM effects, such as HDAC inhibitors and KDM inhibitors, all emphasize the critical role of histone PTMs in the pathogenesis of MS. The amalgamation and crystallization of histone PTMs research and MS promises novel pleiotropic treatment strategies. However, given the potential for off-target potential to cause deleterious effects from HDAC and KDM inhibitors with broad activity, the endeavor to completely understand molecular mechanisms governing histone modifications and their precise molecular targets will hold the key to successfully translate the drug candidates to clinical practice.

## Abbreviations

Ac-CoA: Acetyl coenzyme A
APC: Antigen-presenting cells
BBB: Brain-blood barrier
BRD: Bromodomain
DCs: Dendrite cells
EAE: Experimental autoimmune encephalomyelitis
EBV: Epstein-Barr virus
HATs: Histone acetyltransferaces
HDACs: Histone deacetylases
HLA: Human leukocyte antigen
IFN: Interferon
IL: Interleukin
MHC: Major histocompatibility complex
NAWM: Normal-appearing white matter
PHD: Plant homeodomain
PTMs: Post-translational modifications
TAF1: TATA-binding protein-associated factor-1
TGF: Transforming growth factor
Th: T helper
